# Pace and determinants of implementation of the self-management of well-being group intervention: a multilevel observational study

**DOI:** 10.1186/s12913-019-3891-x

**Published:** 2019-01-25

**Authors:** Daphne Kuiper, Nardi Steverink, Roy E. Stewart, Sijmen A. Reijneveld, Robbert Sanderman, Martine M. Goedendorp

**Affiliations:** 1 Service Desk Clinical Research Office, UMC staff, University Medical Center Groningen, P.O. Box 30.001, AB41, 9700 RB Groningen, The Netherlands; 2 Department of Health Psychology, University Medical Center Groningen, University of Groningen, P.O. Box 196, FA12, 9700 AD Groningen, The Netherlands; 3 Department of Sociology, University of Groningen, Grote Rozenstraat 31, 9712 TG Groningen, The Netherlands; 4 Department of Health Sciences, University Medical Center Groningen, University of Groningen, P.O. Box 196, FA10, 9700 AD Groningen, The Netherlands; 5 Department of Health Sciences, (Community & Occupational Medicine) University Medical Center Groningen, University of Groningen, P.O. Box 196, FA10, 9700 AD Groningen, The Netherlands; 6 Department of Psychology, Health & Technology, University of Twente, P.O. Box 217, 7500 AE Enschede, The Netherlands; 7 Department of Health Science, Faculty of Sciences, Vrije Universiteit Amsterdam, Amsterdam Public Health Research Institute, De Boelelaan 1105, 1081 HV Amsterdam, The Netherlands

**Keywords:** Pace, Implementation, Determinants, Health care, Multilevel-analysis, Older adults, Self-management, Social care, Well-being

## Abstract

**Background:**

When implementing an empirically supported intervention (ESI) arrays of influencing factors operate on the professional and organizational level, but so far dependency between these levels has often been ignored. The aim of this study is to describe the pace and identify determinants of implementation of the Self-Management of Well-being (SMW) group intervention while taking the dependency between professionals and organizations into account.

**Methods:**

Pace of implementation was measured as the time between training of professionals and first use of the SMW intervention in months. Determinants of first use were derived from the Fleuren framework and assessed using web-based questionnaires and telephone interviews. First, univariate analyses, Fisher’s exact tests and t-tests, were performed to identify determinants of first use of the SMW intervention on the individual professional and the organizational level independently. Second, multilevel analyses were performed to correct for the dependency between professionals and organizations. Simple multilevel logistic regression analyses were performed with determinants found significant in the univariate analyses as independent variables, first use as dependent variable, professionals entered in the first level, and organizations in the second level.

**Results:**

Forty-eight professionals from 18 organizations were trained to execute the SMW intervention. Thirty-two professionals achieved first use, at a mean pace of 7.5 months ± 4.2. Determinants on the professional level were ‘ownership’, ‘relative advantage’, ‘support from colleagues’ and ‘compatibility’. Determinants on the organizational level were ‘organizational size’ and ‘innovation-task orientation fit’. Multilevel analysis showed that ‘compatibility’, a factor on the professional level, was the only significant determinant contributing to first use in the multilevel model.

**Conclusions:**

This implementation study revealed a strong dependency between professionals and organizations. Results showed that a majority of professionals used the SMW intervention in about 8 months. When the dependency between professionals and organization was taken into account, the professionals’ perception of compatibility was the only remaining determinant of implementation on the professional level. Organizational size and managers’ perception of ‘innovation-task orientation fit’ were determinants of implementation on the organizational level. It is advisable to discuss the compatibility between new and current tasks among managers and professionals before adopting a new intervention.

**Electronic supplementary material:**

The online version of this article (10.1186/s12913-019-3891-x) contains supplementary material, which is available to authorized users.

## Background

The gap between science and practice is well-known [1, 2]. Many empirically supported interventions (ESIs) are neither adopted nor successfully implemented in practice [3, 4]. Moreover, the ones that do succeed to ‘bridge the gap’, take many years to do so [5, 6], but then still the complex interplay of determinants and stakeholders is not yet understood. It has been established that Self-Management of Well-being (SMW) interventions^1^ improve self-management ability and well-being, and reduce loneliness in older adults [7–11]. When adopted and implemented in health and social care settings, the SMW interventions may have the potential to constrain the accumulating prevalence of social isolation [12], depression [13], and inactivity [14] in older adults. Therefore, the current study aimed to investigate the implementation of the SMW group intervention in health and social care, with special focus on the pace of this process and the dependency between individual and organizational determinants in this process.

It is known that new interventions often take long to be implemented in practice, even though time effectiveness has been identified as a priority [6, 15]. Reasons to transit from adoption to implementation in ‘a relatively quick manner’ is important, because one-third of the newly learned competencies in professionals will be lost after one year [16]. Furthermore, organizations perceive implementation as a return on their investment in having professionals trained. This return diminishes when professionals fail to attain first use of a new intervention [17]. The rule of thumb is that it takes between two and six months to attain first use [18]. Since the pace of implementation of the SMW group intervention is not yet known, it is subject of investigation in this study.

It is known that a wide array of facilitating and impeding factors affect implementation. Literature reviews produce comprehensive lists, ranging from 23 up to 50 factors [1, 19–23]. Some also acknowledging that different factors operate at different stakeholder levels, such as professionals, organizations, and financial-political contexts [23]. However, not many studies take the interdependencies between these stakeholders into account when investigating factors affecting implementation.

Although many implementation models exist that distinguish various stakeholder levels [24], to our knowledge, hardly any models exist that provide clear directions how to empirically investigate determinants of the implementation process. An exception is the Fleuren framework, which provides detailed descriptions to determine which stakeholder’s factors are important at what stage of the implementation process [21].

First, the Fleuren framework distinguishes four stages: orientation, adoption, implementation and continuation [21]. The transition from adoption to implementation has been recognized to be the most challenging [18]. Professionals have to become proficient in applying new just acquired skills, and organizations have to be or become efficient in allocating time and resources to the new way of doing things [18]. Therefore, the focus of this study is on exactly this stage in the implementation process, i.e. the stage where professionals and their organization transit from adoption to first use of the SMW intervention.

Second, the Fleuren framework identifies determinants of implementation on both the professional and organizational level. It is very important to realize that professional and organization levels are not independent [25]. Proficient professionals might not start using a new intervention if there are organizational barriers. Vice versa, a facilitating organization will not start using a new intervention when there are professional barriers. Therefore, this study will not only investigate which determinants play a role on either the professional or the organizational level when implementing the SMW group intervention, but also which factors remain determinants when taking the nested structure of professionals in their organizations into account.

### Study aims

The aims of this study are to investigate the pace of implementation of the SMW group intervention in health and social care organizations, and to identify determinants of implementation while taking the dependency between professionals and organizations into account. Three research questions will be addressed: 1) How many of the professionals trained, realize first use of the SMW group intervention and at what pace? 2) What are the determinants of first use in: 2a) professionals, and 2b) organizations? 3) What are the determinants of first use when the nesting of professionals in organizations is taken into account?

## Method

### Study design

The current study is part of a larger implementation project that aimed to identify the determinants of use of the SMW interventions in health and social care organizations. The study protocol of this larger project has been described in detail elsewhere [26].

In short, the project entails four overlapping project phases and started in April 2010. In project phase 1 (month 0–12) the aim was to motivate at least 15 organizations to adopt the SMW group intervention. In project phase 2, between May 2010 and March 2011, professionals (two per organization) were trained to perform the SMW group intervention (see training). In project phase 3 the trained professionals started implementing the SMW group intervention by recruiting older adults aiming to reach 400 participants. Data collection waves were scheduled in month 12–15 (T1), month 24–26 (T2) and month 36–39 (T3). In phase 4 data analyses were executed. The current study is based on the data collected at T2 between April and June 2012.

### Study sample

The study sample consists of participants at two hierarchical levels: the professional and the organizational level. The professional level was represented by trained SMW professionals, and the organizational level was represented by managers closest to the SMW professionals.

### Setting

The study was performed in the northern part of the Netherlands. Various health and social care organizations in that region contribute to the execution of the nationwide Social Support Act (2007), which prescribes that vulnerable older adults and other vulnerable citizens need to be supported to recapture or maintain their ability to manage their own well-being. The objectives of the SMW group intervention correspond largely to the purposes of this Act, which makes health and social care organizations suitable settings for implementing the SMW intervention. Health care organizations interested in participating were predominantly home-care organizations, employing public health nurses aimed at supporting older adults in their direct living environment (home and/or neighborhood). The interested social care organizations were predominantly organizations employing social service or social group workers specialized in executing community-based services for specific vulnerable target groups such as lonely older adults.

### Recruitment

In project phase 1, from April 2010, an invitation to participate in the implementation project was disseminated among all formal health and social care organizations in the northern part of the Netherlands. For interested health and social care organizations, SMW workshops were given for professionals and managers of these organizations.

### The SMW intervention

The SMW group intervention is designed for socially vulnerable women, aged > 55 years, who subscribe individually, and are physically capable of travelling to a group location. It is specifically designed for women, because in general these kinds of interventions require gender-specific topics, wording and assignments and the need for such support was largest among them. The SMW group intervention is theory-driven, applying the theory of Self-Management of Well-being (SMW theory) [27, 28]. This theory postulates that if people have good self-management abilities – that is, skills enabling them to adequately handle their important physical and social resources – they are able to maintain a higher level of overall well-being. The SMW group intervention has proven to be effective in enhancing self-management ability and well-being, and reducing loneliness [8, 11].

The SMW group intervention consists of six consecutive group meetings (8–12 participants) and one booster session after three months. Each session takes 2½ hours and is being supervised by two trained professionals. Each meeting focuses on one or more of the six self-management abilities identified by the SMW theory [27, 28]. The six self-management abilities are: 1) taking initiatives; 2) being self-efficacious; 3) investing; 4) having a positive outlook; 5) ensuring multi-functionality in resources; 6) ensuring variety in resources. The women are taught to apply these abilities to the five dimensions of well-being, summarized by the Dutch acronym *GLANS* [Dutch for gleam or gloss]. In the acronym G stands for *Gemak and Gezondheid* [easy living and health: Comfort], L for *Leuke bezigheden en Lichamelijke activiteit* [pleasant and physical activity: Stimulation], A for *Affectie* [giving and receiving love and affection: Affection], N for *Netwerk* [social network contacts: Behavioral confirmation], and S for *Sterke punten* [strengths: Status]). For a detailed description of the intervention see Kremers et al., 2006 [8].

### Training professionals

The professionals of the health and social care organizations that adopted the SMW group intervention followed the standardized SMW training of 2½ days taught by two qualified SMW trainers. Professionals were admitted to the training under three conditions (1) being female, because the SMW group intervention participants would all be women, (2) being currently employed in a formal health and/or social organization, and (3) registering together with one or more female colleagues from the same organization.

The training focused on knowledge transfer regarding SMW theory and on practicing skills. Skills needed to guide and supervise the SMW group intervention were trained by means of role-play and feedback. Additional instructions were given on the intervention materials (manual and workbook for participants) and the availability of support from the research team by means of the implementation toolkit, website and site-visits. The professionals were certified as SMW teachers after completion of the training.

### Adaptation Fleuren framework to fit the SMW implementation study

**Fig. 1:**
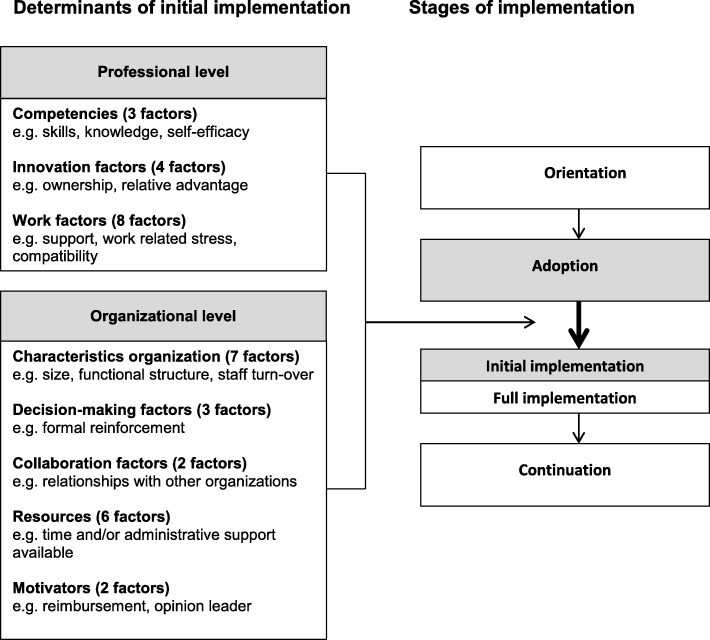
Framework of factors affecting first use of the Self-Management of Well-being group intervention (adapted from Fleuren et al., 2004 and 2010)

The data collection is based on the original Fleuren framework, which identifies 50 determinants on various stakeholder levels and stages of implementation [21]. However, we needed to adapt this general framework for the study at hand, i.e., for the case of the SMW intervention, because not all levels and determinants fitted our study. For example, we removed the originally defined level of the innovation and attributed its factors (e.g. ‘clearness procedures’ and ‘appealing to use’) to the individual professional level, because these factors would need to be assessed by the professionals that execute the SMW intervention (innovation). Additionally, we renamed some levels and factors. For example, ‘users’ in the Fleuren framework we referred to as the ‘professionals’, and ‘patients’ were ‘clients’ in the current study. This adaptation process of the Fleuren implementation framework is described in detail elsewhere [26]. The resulting implementation framework for the current study identifies four stages of implementation and 35 determinants on the professional and the organizational level (see Fig. 1). On the professional level, 15 factors were sorted into three clusters: competencies, innovation and work situation. On the organizational level 20 factors were sorted into five clusters: characteristics of the organization, decision-making, collaboration, resources and motivators.

### Data collection

The current study is based on the data collected at T2. At that time, professionals have had at least 13 months to realize implementation and use of the SMW group intervention. Three types of data were collected by the research team: 1) observational data: training dates, and starting dates of the SMW intervention were recorded; 2) web-based questionnaires were filled out by professionals, and 3) structured telephone interviews were held among managers.

All professionals were contacted, and two reminders were sent when they did not respond. Five professionals did not respond, because of compulsory redundancy (*n* = 2), sick leave (n = 2), and technical failure (*n* = 1). Two of the non-responding professionals were users and three were non-users of the SMW intervention. All managers (100% response) of the 18 organizations were willing to participate in the telephone interview.

### Dependent variables

The ‘pace’ of implementation is expressed as the time in months between having completed the training and first use of the SMW intervention by professionals. ‘Implementation’ on the professional level is assessed as ‘first use’ (yes/no) of the SMW group intervention by the professionals. Implementation on the organizational level is expressed in first use ratio (i.e. the number of SMW-trained professionals using the SMW intervention at T2 divided by the number of all SMW-trained professionals in the organization).

### Independent variables

To assess the determinants of implementation of the SMW group intervention in health and social care organizations, the Fleuren framework [21] was adapted for the current study. Next, the checklist for determinants of innovations in health care organizations, published by Fleuren in 2010 [29], was used to operationalize factors (determinants) into items. Fifteen factors on the professional level were assessed with 31 items in three clusters: competencies (3 factors, 5 items), innovation (4 factors, 7 items) and work situation (8 factors, 19 items). On the organizational level 20 factors were assessed with 23 items in five clusters: characteristics of the organization (7 factors, 10 items), decision-making (3 factors, 3 items), collaboration (2 factors, 2 items), resources (6 factors, 6 items) and motivators (2 factors, 2 items). The predefined factors from the framework were translated into one or more closed questions per factor. For example, the factor “compatibility” on the professional level (described by Fleuren (2010) [29] as “to what extent does the care provider view the innovation as matching his/her job description)” is operationalized by two items: (1) “Is preparing and executing the SMW group intervention part of your formal task description?” (2) “Do you have enough time to prepare and execute the SMW group intervention?” If Cronbach’s alpha was > 0.7, items measuring the same factor were combined into a scale [30]. Single items and scales were dichotomized and given the value ‘1’ for facilitating first use, and value ‘0’ when impeding first use. The questionnaire, as well as the scales and recoding of scores, is described in Additional file 1.

### Data analysis

The observational data, the data from the web-based questionnaire, and those from the telephone interviews were entered using the IBM SPSS statistics 20 program. Descriptive statistics on the characteristics of the study sample are reported as means, standard deviations and ranges in case of continuous variables, and as the absolute numbers and percentages in case of discrete variables. Descriptive statistics on the pace of implementation are reported as mean, standard deviation and range in months.

Univariate analyses were performed to identify the determinants of first use on the professional and on the organizational level. Fisher’s exact tests (one-sided with a significance level set at *p* < 0.05) were used to test which factors were significantly associated with first use (yes/no) on the professional level. Independent samples t-tests were used to test which factors were significantly associated with the mean first user ratio (% of users per organization) on the organizational level.

Multilevel analyses were performed to identify the determinants of first use in context (professionals nested in organizations). Significant determinants of first use found in univariate analyses were selected and imported in Mplus, version 7.1 [31]. Subsequently, simple multilevel logistic regression analyses [32] were performed with the data of 43 professionals (level 1) nested in the data of 18 organizations (level 2) and first use (yes/no) as the dependent variable. For each factor an unadjusted analysis was performed.

## Results

### Characteristics study sample

In total 48 professionals from 18 different organizations were trained and certified to execute the SMW group intervention. The average number of professionals trained per organization was 2.7 (range 1–5). In total, 18 managers, one from each organization, participated in the study.

All professionals were female and most (*n* = 29) were social service workers. Most managers (*n* = 12) were also female, and in a lower or middle management job. The characteristics of the professionals and managers are described in Table 1.

**Table 1:** Characteristics study sample

Professionals (*n* = 48)		mean	(sd)	range	*n*	(%)
Gender	female				48	(100)
	male				0	(0)
Age	years	50.4	(9.0)	26–62		
Work hours^a^	hours per week	24.1	(5.6)	8–36		
Job description	social service worker				29	(60)
	social group worker				8	(17)
	public health nurse				3	(6)
	other (e.g. psychomotor therapist)				8	(17)
Work setting	health organization				9	(19)
	social work organization				39	(81)
Managers (*n* = 18)		mean	(sd)	range	n	(%)
Gender	female				12	(67)
	male				6	(33)
Age	years	52.9	(5.9)	39–63		
Work hours^a^	hours per week	31.2	(6.0)	20–36		
Job description	higher management				4	(22)
	middle management				8	(45)
	lower management				6	(33)
Work setting	health organization				3	(17)
	social work organization				15	(83)

*sd* standard deviation

^a^Work hours according to employment contract

### Pace of implementation of the SMW group intervention

Two third of the SMW professionals (32/48) achieved first use of the SMW group intervention at T2. The mean pace at which they achieved first use was 7.5 months (SD 4.2 months, range 1–17 months). Six percent (3/48) of the professionals achieved first use within 3 months, 29% (14/48) between 3 and 6 months, 23% (11/48) between 7 and 12 months and in 8% (4/48) it took longer than a year.

### Determinants of first use of the SMW group intervention on the professional level

In Table 2 the results on the professional level are shown.

**Table 2:** Factors on the professional level affecting first use

	Use (*n* = 30)		No use (*n* = 13)		*p*-value
	*n*	(%)	*n*	(%)	
Factors related to competencies professional					
Skills					
1 = prior experience working with groups of older adults	26	(72.2)	10	(27.8)	.561
0 = no prior experience working with groups of older adults	4	(66.7)	2	(33.3)	
Knowledge					
1 = higher education	27	(71.1)	11	(28.9)	.482
0 = vocational education	3	(60.0)	2	(40.0)	
Self-efficacy					
1 = confidence in recruiting, organizing and supervising the group	17	(85.0)	3	(15.0)	.064
0 = no confidence in recruiting, organizing and supervising the group	13	(59.1)	9	(40.9)	
Factors related to the innovation					
Ownership					
1 = feeling responsible for SMW group intervention implementation	25	(80.6)	6	(19.4)	.036*
0 = not feeling responsible for SMW group intervention implementation	5	(45.5)	6	(54.5)	
Clearness procedures					
1 = SMW manual is clear	30	(73.2)	11	(26.8)	.286
0 = SMW manual is not clear	0	(0.0)	1	(100.0)	
Relative advantage					
1 = advantage	21	(87.5)	3	(12.5)	.010*
0 = no advantage	9	(50.0)	9	(50.0)	
Appealing to use					
1 = appealing	22	(66.7)	11	(33.3)	.190
0 = not appealing	8	(88.9)	1	(11.1)	
Factors related to work situation					
Support from SMW-colleagues					
1 = positive actions	27	(79.4)	7	(20.6)	.031*
0 = no positive actions	3	(37.5)	5	(62.5)	
1 = positive cooperation (missing *n* = 11)	28	(93.3)	2	(6.7)	.877
0 = no positive cooperation	2	(100.0)	0	(0.0)	
Support from other colleagues					
1 = positive attitude	25	(75.8)	8	(24.2)	.216
0 = no positive attitude	5	(55.6)	4	(44.4)	
1 = positive actions	15	(71.4)	6	(28.6)	.633
0 = no positive actions	15	(71.4)	6	(28.6)	
Support from supervisor					
1 = positive attitude	28	(75.7)	9	(24.3)	.131
0 = no positive attitude	2	(40.0)	3	(60.0)	
1 = positive actions	17	(77.3)	5	(22.7)	.296
0 = no positive actions	13	(65.0)	7	(35.0)	
Support from higher management					
1 = positive attitude	23	(76.7)	7	(23.3)	.207
0 = no positive attitude	7	(58.3)	5	(41.7)	
1 = positive actions (missing *n* = 9)	9	(75.0)	3	(25.0)	.351
0 = no positive actions	19	(86.4)	3	(13.6)	
Modelling					
1 = stimulated by implementation success of other organizations	18	(72.0)	7	(28.0)	.481
0 = not stimulated by implementation success of other organizations	12	(66.7)	6	(33.3)	
Factors related to work situation (continued)					
Innovation task-orientation fit					
1 = fit between innovation and needs older adults	29	(72.5)	11	(27.5)	.231
0 = no fit between innovation and needs older adults	1	(33.3)	2	(66.7)	
1 = fit between innovation and perceived task professional	24	(70.6)	10	(29.4)	.589
0 = no fit between innovation and perceived task professional	6	(75.0)	2	(25.0)	
Work related stress					
1 = no overtime work	15	(68.2)	7	(31.8)	.540
0 = overtime work	15	(71.4)	6	(28.6)	
1 = no sick leave	23	(69.7)	10	(30.3)	.654
0 = sick leave	7	(70.0)	3	(30.0)	
1 = satisfied with job	28	(71.8)	11	(28.2)	.350
0 = not satisfied with job	2	(50.0)	2	(50.0)	
1 = no work pressure	2	(66.7)	1	(33.3)	.671
0 = work pressure	28	(70.0)	12	(30.0)	
Compatibility					
1 = compatible	14	(93.3)	1	(6.7)	.019*
0 = not compatible	16	(59.3)	11	(40.7)	

* *p* -values < .05 were significant

*SMW*  Self-management of Well-being

None of the three factors related to competencies of the professional was significantly associated with first use of the SMW group intervention (all *p* > .064). However, two of the four factors related to the innovation, were significantly associated with first use of the SMW group intervention. The majority of the SMW professionals felt responsible for implementation of the SMW group intervention (‘ownership’) and those who did feel ownership achieved first use more often than those who did not feel ownership (*p* = .036). The majority of the SMW professionals also perceived using the SMW group intervention as advantageous for themselves (‘relative advantage’). Those who perceived relative advantage achieved first use more often than those who did not perceive relative advantage (*p* = .010).

Of the eight factors related to the work situation, two showed to be significantly associated with first use of the SMW group intervention. The majority of the SMW professionals reported positive actions by their direct SMW colleagues (‘support’). Those who did feel supported achieved first use more often than those who did not feel supported (*p* = .031). A minority of SMW professionals reported that implementing the SMW group intervention was part of their formal task description, and that they had enough time to prepare and execute the intervention (‘compatibility’). Those who did report that implementing the SMW group intervention was compatible with other designated tasks, achieved first use more often than those who reported non-compatibility (*p* = .019).

### Determinants of first use of the SMW group intervention on the organizational level

In Table 3 the results on the organizational level are shown.

**Table 3:** Factors on the organizational level (*n* = 18) affecting mean first user ratio

		*n*	Mean % of users per organization	*p*-value
Factors related to characteristics organization				
Size				
Size organization				
1 = large (> 150 employees)		8	.97	.018*
0 = small (< 150 employees)		10	.52	
Size unit				
1 = large (> 10 Ftu)		8	.72	.750
0 = small (< 10 Ftu)		8	.65	
Structure				
Functional structure				
1 = task oriented		11	.72	.814
0 = output oriented		6	.67	
Setting				
1 = social work		15	.68	.384
0 = health		3	.92	
Staff turn-over				
1 = low		3	.83	.616
0 = high		15	.69	
Staff capacity				
1 = sufficient		6	.62	.523
0 = insufficient		12	.76	
Innovation task-orientation fit				
1 = fit between innovation and needs older adults		16	.75	.453
0 = no fit between innovation and needs older adults		2	.50	
1 = fit between innovation and view management		15	.79	.008*
0 = no fit between innovation and view management		2	.00	
Expectations cooperation target group				
1 = positive		1	1	.544
0 = not positive		16	.75	
Expectations satisfaction target group				
1 = positive		13	.71	.376
0 = not positive		2	1	
Factors related to decision-making				
Decision making process and procedures				
1 = both professionals and management participated		7	.89	.164
0 = professionals or management decided (bottom-up or top-down)		11	.61	
Hierarchical structure				
1 = short communication channels (low formalization)		15	.67	.211
0 = long communication channels (high formalization)		3	1	
Formal reinforcement				
1 = formal reinforcement (incorporated in annual report)		14	.78	.250
0 = no formal reinforcement (not incorporated in annual report)		4	.50	
Factors related to collaboration				
Relationships with other organizations				
1 = outreaching		17	.76	
0 = introvert		0	0	
Nature of collaboration internally				
1 = good collaboration		16	.68	.327
0 = poor collaboration		2	1	
Factors related to resources				
Available expertise				
1 = much expertise		14	.64	.189
0 = little expertise		3	1	
Logistical procedures				
1 = well arranged		13	.75	.614
0 = badly arranged		5	.63	
Other (material) resources available				
1 = available		12	.65	.318
0 = not available		6	.86	
Administrative support available				
1 = available		11	.69	.804
0 = not available		7	.75	
Time available				
1 = time available		7	.71	.979
0 = no time available		11	.72	
Coordinator available				
1 = coordinator available		16	.75	.453
0 = no coordinator available		2	.50	
Factors related to motivators				
Reimbursement				
1 = reimbursement		2	.87	.589
0 = no reimbursement		16	.70	
Opinion leader				
1 = available		11	.75	.695
0 = not available		7	.67	

* *p*-values < .05 were significant

*FTU* Functional Task Unit

Two of the seven organizational characteristics showed to be significantly associated with the mean first user ratio. In organizations with more than 150 employees (‘size organization’) the mean first user ratio was significantly higher than in their smaller counterparts (*p* = .018). Additionally, in organizations where the SMW group intervention complied with the task orientation of the management (i.e. ‘innovation - task orientation fit’) the mean first user ratio was significantly higher than in organizations where there was no fit (*p* = .008). All other organizational characteristics did not yield statistically significant results (see Table 3).

### Determinants of first use of the SMW group intervention when professionals are nested in their organizations

The intraclass correlation coefficient for the empty model was 0.8. Based on the previous single level analyses, six significant factors were identified. For each factor a simple multilevel logistic regression analysis was performed. Multilevel modelling was not possible for one factor, namely ‘innovation - task orientation fit’, because of empty cells in the joint distribution of professionals nested in organizations. These analyses showed that ‘compatibility’ (professionals’ perception that implementing the SMW group intervention was compatible with other designated tasks), was the only significant factor contributing to first use on the professional level in the multilevel model (odds ratio 59.8; 95% confidence interval 1.17–3044; *p* = .041).

## Discussion

This study showed that two out of three trained professionals achieved first use of the SMW group intervention in about eight months. Four determinants of using the SMW intervention were identified on the professional level. When the nested structure of professionals within organizations was taken into account, compatibility on the professional level, remained the only significant determinant of first use on the professional level, confirming the importance of organizational dependency. Organizational size and managerial innovation-task orientation fit were factors on the organizational level that determined first use of the SMW group intervention.

To our knowledge not many implementation studies report utilization rate and pace of implementation. However, the utilization-rate of 67% found in the current study is similar to another community-based intervention; the rate in this Positive Parenting Program (TripleP) was 63 and 70% [17, 33, 34]. The mean pace at which SMW professionals realized first use of the SMW group intervention was 7.5 months. This is a little bit slower than the 2–6 months, as indicated by Fixsen et al., 2007 [18], but much quicker than the start-up time reported in other studies. Implementation of a Colorectal Cancer Screening Demonstration program took 9–11 months to start-up [35]. Implementation of AHA guidelines took 13 months [36], while implementation of a software program in laboratories took 1.8 years [37]. Although, the above mentioned implemented interventions are different in nature, it seems that the utilization rate and the pace of implementation of the SMW group intervention in health and social care is quicker. So overall, the pace of implementation of the SMW group intervention was successful.

Four determinants of first use could be identified on the professional level when the dependency of organizations was ignored. The chance of a professional realizing first use of the SMW group intervention seemed to be determined by the extent to which she perceives ‘ownership’, ‘relative advantage’, ‘support’ and/or ‘compatibility’. However, when the nested structure of professionals in organizations was taken into account, results showed that professionals’ perceived ‘compatibility’ was the only remaining significant key determinant. This means that independently of the organization in which professionals work, compatibility is an important factor of professionals that determines use of the SMW intervention. Compatibility seems an important facilitating factor for implementation, as confirmed by other studies. For example, it was found that the innovation needs to fit the work and routines of healthcare professionals [38] and Van der Stege [39] found that incompatibility of own goals with intervention goals was a barrier of implementation.

‘Ownership’, ‘relative advantage’ and ‘support perceived by the professional’ were no longer significant determinants of SMW intervention first use, when the dependency of the organization was taken into account. This means that the organization, in which the professionals work, has a stronger influence on whether or not the SMW intervention will be used than these three determinants on the professional level.

Our results showed that ‘organizational size’ and ‘managerial innovation-task orientation fit’ were determinants of mean first user ratio on the organizational level. However, we didn’t find any significant organizational determinants of first use of the SMW group intervention on the professional level. Regarding this finding, it should be noted that first use on the professional level and mean first user ratio, are different outcome measures, with the latter being a collective measure. It remains speculative why larger organizations realized use of the SMW intervention more often than smaller organizations, but it could be that, for example, the availability of a PR department in larger organizations might be a facilitating factor. Nevertheless, the finding that ‘managerial innovation - task orientation fit’ was also a significant determinant, indicates that compatibility of the SMW intervention with current tasks was important for both professionals and managers to start using the SMW intervention.

The strengths of this study are that it is an observational study in health and social care settings that applies both a predefined implementation framework and a multilevel approach to identify determinants of successful implementation of a new evidence-based intervention. Though the use of frameworks in implementation sciences has significantly increased over the past decades [24, 40], the use of multilevel analysis techniques has primarily been confined to health care professionals’ behavior or behavioral intentions to use information technology [41, 42] or research utilization in general [43, 44]. To our knowledge, multi-level analyses investigating model-based determinants of implementation in health and social care settings are scarce (for an exception see: [45]).

Despite these strengths some limitations should be noted. First, the sample size was a limitation. Even though we had more than 10 groups as recommended for multilevel logistic regression analysis [32], our sample was too small for multivariate testing in the multilevel model. Moreover, the large odds ratio and wide 95% confidence interval of the multilevel analysis could be attributed to the small sample size as well, and therefore, the results should be interpreted with caution. Additionally, we may have missed some significant single level determinants, because of the small sample size. For example, ‘self-efficacy’, a well-known factor on the professional level from other studies [21, 23, 46], might also have proven to be significant when many more professionals and organizations had participated in the project. Although we succeeded to engage more organizations and professionals than planned, implementation research is typically beset by a “small N” problem [15].

Second, although we assessed determinants on the level of the professional and the organization, other contextual factors could be important too. According to the original model of Fleuren et al. (2004) factors on the level of the financial political context, such as rules and legislation, and financial resources, are relevant too [21]. In our larger project [26], we did consider these factors, but due to the restricted scope of the project, we were not able to get sufficient and valid data on these factors. Additionally, it should be noted that this study was performed in four provinces in the Northern part of the Netherlands, so our results might not apply to other regions in the Netherlands, nor to other countries.

Third, the items to measure the potential determinants based on the Fleuren framework [21] have been self-constructed and have not yet been validated. Therefore, it is unknown whether the factors are measured reliably and validly. In the meantime, Fleuren et al. (2014) have developed the MIDI-instrument [47], but this instrument was not available by the time we had to assess the factors. Moreover, the MIDI instrument also needs validation.

Fourth, a control group could have been of added value to the design. An ideal design would have included organizations with similar baseline characteristics that did not adopt the SMW intervention. However in practice, this would be very hard to realize.

Furthermore, one could reason that the level of implementation might be overestimated, because it has been shown that implementation can deteriorate over time [23], and we assessed use early in the project, two years after the start of the project. However, we don’t expect that the level of implementation was overestimated, because some of the professionals were trained relatively late in the project and had relatively limited time to start using the SMW intervention. To get a full understanding of the process of implementation of innovations in health and social care, it is needed to collect data over multiple time points [23].

Despite these limitations, this study offers useful directions for implementation practice. Given the outcome that, regardless of organizational size and culture, compatibility with other designated tasks on the individual professional level is the key determinant in the first toilsome stage from adoption to initial implementation, it is important that managers create an environment that professionals perceive as encouraging for implementation [48]. As also advised by Van der Kleij et al. [49], emphasis should be placed on limiting the number of prescribed activities and allocating sufficient time to get experienced with the innovation. Therefore we recommend for clinical practice, that managers and administrators of adopting organizations should not only properly reserve formal and operational time for intended users, but also fine-tune allocated time with professionals during the process. Fine-tuning should not only encompass telling the professional what they can start doing new, but also telling them what they can stop doing the conservative way.

## Conclusions

Forty-eight professionals from 18 health or social care organizations adopted the SMW group intervention. Thirty-two of them achieved first use at a pace of almost 8 months. The current study showed that ‘ownership’, ‘relative advantage’, ‘support from colleagues’ and ‘compatibility’ were determinants of implementation on the professional level, while ‘organizational size’ and ‘innovation - task orientation fit’ were determinants on the organizational level. Multilevel analyses, taking the dependency between professionals and organizations into account, showed that the professionals’ perception of compatibility, i.e. whether the SMW intervention was compatible with other designated tasks, was the main determinant of implementation on the professional level.

## Additional file

Additional file 1: Questionnaire for Professionals (XLSX 26 kb)

## Abbreviations

ESI: Empirically Supported Intervention
SMW: Self-Management of Well-being

## Footnotes

1 In former publications the SMW interventions were also referred to as “GRIP and GLEAM (G&G) interventions”.
